# Improved variant discovery through local re-alignment of short-read next-generation sequencing data using SRMA

**DOI:** 10.1186/gb-2010-11-10-r99

**Published:** 2010-10-08

**Authors:** Nils Homer, Stanley F Nelson

**Affiliations:** 1 Department of Computer Science, University of California - Los Angeles, Boelter Hall, Los Angeles, CA 90095, USA; 2 Department of Human Genetics, David Geffen School of Medicine, University of California - Los Angeles, 695 Charles Young Drive South, Los Angeles, CA 90025, USA; 3 Current address: Ion Torrent, Life Technologies, 7000 Shoreline Court, South San Francisco, CA 94080, USA

## Abstract

A primary component of next-generation sequencing analysis is to align short reads to a reference genome, with each read aligned independently. However, reads that observe the same non-reference DNA sequence are highly correlated and can be used to better model the true variation in the target genome. A novel short-read micro re-aligner, SRMA, that leverages this correlation to better resolve a consensus of the underlying DNA sequence of the targeted genome is described here.

## Background

Whole-genome human re-sequencing is now feasible using next generation sequencing technology. Technologies such as those produced by Illumina, Life, and Roche 454 produce millions to billions of short DNA sequences that can be used to reconstruct the diploid sequence of a human genome. Ideally, such data alone could be used to *de novo *assemble the genome in question [1-6]. However, the short read lengths (25 to 125 bases), the size and repetitive nature of the human genome (3.2 × 10^9 ^bases), as well as the modest error rates (approximately 1% per base) make such *de novo *assembly of mammalian genomes intractable. Instead, short-read sequence alignment algorithms have been developed to compare each short sequence to a reference genome [7-12]. Observing multiple reads that differ similarly from the reference sequence in their respective alignments identifies variants. These alignment algorithms have made it possible to accurately and efficiently catalogue many types of variation between human individuals and those causative for specific diseases.

Because alignment algorithms map each read independently to the reference genome, alignment artifacts could result, such that SNPs, insertions, and deletions are improperly placed relative to their true location. This leads to local alignment errors due to a combination of sequencing error, equivalent positions of the variant being equally likely, and adjacent variants or nearby errors driving misalignment of the local sequence. These local misalignments lead to false positive variant detection, especially at apparent heterozygous positions. For example, insertions and deletions towards the ends of reads are difficult to anchor and resolve without the use of multiple reads. In some cases, strict quality and filtering thresholds are used to overcome the false detection of variants, at the cost of reducing power [13]. Since each read represents an independent observation of only one of two possible haplotypes (assuming a diploid genome), multiple read observations could significantly reduce false-positive detection of variants. Algorithms to solve the multiple sequence alignment problems typically compare multiple sequences to one another in the final step of fragment assembly. These algorithms use graph-based approaches, including weighted sequence graphs [14,15] and partial order graphs [16,17]. Read re-alignment methods also have been developed [2,18] for finishing fragment assembly but have not been applied to the short reads produced by next generation sequencing technologies.

In this study, a new method to perform local re-alignment of short reads is described, called SRMA: the Short-Read Micro re-Aligner. Short-read sequence alignment to a reference genome and *de novo *assembly are two approaches to reconstruct individual human genomes. Our proposed method has the advantage of utilizing previously developed short-read mapping as the input, coupled with an assembly-inspired approach applied over discrete small windows of the genome whereby multiple reads are used to identify a local consensus sequence. The proposed method overcomes problems specific to alignment and genome-wide assembly, respectively, with the former treating reads independently and the latter requiring nearly error-free data. Unlike *de novo *assembly, SRMA only finds a novel sequence variant if at least one read in the initial alignment previously observed this variant. *De novo *assembly algorithms, such as ABySS and Velvet [1-3,5,6,19], could be applied to reads aligned to local regions of the genome to produce a local consensus sequence, which would need to be put in context to the reference sequence. This approach may still show low sensitivity due to the moderate error found in the data and has not been implemented in practice. For this reason, an important contribution of SRMA is to automate the return of alignments for each read relative to the reference.

SRMA uses the prior alignments from a standard sequence alignment algorithm to build a variant graph in defined local regions. The locally mapped reads in their original form are then re-aligned to this variant graph to produce new local alignments. This relies on the presence of at least one read that observes the correct variant, which is subsequently used to inform the alignments of the other overlapping reads. Observed variants are incorporated into a variant graph, which allows for alignments to be re-positioned using information provided by the multiple reads overlapping a given base. We demonstrate through human genomic DNA simulations and empirical data that SRMA improved sensitivity to correctly identify variants and to reduce false positive variant detection.

## Results and discussion

### Local re-alignment of simulated data

To assess the performance of local re-alignment on a dataset with a known diploid sequence, two whole genome human re-sequencing experiments were simulated (see Materials and methods) to generate 1 billion 50 base-paired end reads for a total of 100 Gb of genomic sequence representing a mean haploid coverage of 15 × for either Illumina or ABI SOLiD data. SNPs, small deletions, and small insertions were introduced to provide known variants and test improvements of SRMA for their discovery genome-wide, as described in the Materials and methods. The data were initially aligned with BWA (the Burrows Wheeler Alignment tool) [9] and then locally re-aligned with SRMA. For ABI SOLiD data, SRMA is able to utilize the original color sequence and qualities in their encoded form. However, BWA does not retain this information, so that only the decoded base sequence and base qualities produced by BWA were used by SRMA. The aligned reads were used for variant calling before and after local SRMA re-alignment by implementing the MAQ consensus model within SAMtools [10,20].

**Figure 1:**
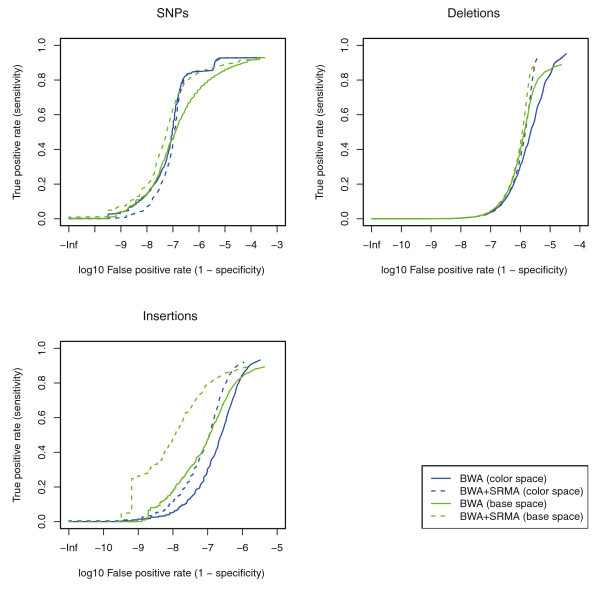
**Local re-alignment receiver operator characteristic curves for simulated human genome re-sequencing data**. A synthetic diploid human genome with SNPs, deletions, and insertions was created from a reference human genome (hg18) as described in main text. One billion paired 50-mer reads for both base space and color space were simulated from this synthetic genome to assess the true positive and false positive rates of variant calling after re-sequencing. An increasing SNP quality filter was used to generate each curve. The simulated dataset was aligned with BWA (v.0.5.7-5) with the default parameters [9]. The alignments from BWA and SRMA were variant called using the MAQ consensus model implemented in SAMtools (v.0.1.17) using the default settings [10,20]. For the simulated datasets, the resulting variant calls were assessed for accuracy by comparing the called variants against the known introduced sites of variation. The BWA alignments were locally re-aligned with SRMA with variant inclusive settings (*c *= 2 and *p *= 0.1).

In Figure 1, we plot receiver operator characteristic (ROC) curves for the detection of the known SNPs, deletions, and insertions. For all types of variants, performing local re-alignment with SRMA greatly reduced the false-positive rate while maintaining the same level or increased sensitivity prior to SRMA. The false-positive reduction is more evident for indels, largely due to the ambiguity of placing indels relative to the reference sequence based on the initial gapped alignment. At this level of mean coverage, false discovery can be reduced to a rate of 10^-6 ^for all variants while maintaining >80% power (sensitivity). We note that because inserted bases are directly observed, insertions are more powerfully corrected to the actual sequence relative to deletions. This may help explain the relatively greater improvement in the false positive rate for insertions over deletions at comparable sensitivities.

These simulations assumed ideal conditions: no genomic contamination, a simple error model with a modest uniform error rate, and a simplification that includes only a subset of all possible variants (SNPs, deletions, and insertions). Nevertheless, the false positive rates achieved after variant calling with no filtering criteria applied is striking and indicates that local re-alignment can be a powerful tool to improve variant calling from short read sequencing. Longer insertions (>5 bp) are not sufficiently examined in the simulation model. However, we note that longer indels are supported by SRMA, but SRMA requires that the initial global alignment permits the sensitive alignment of reads with longer indels to the approximate correct genomic position.

### Local re-alignment of empirical data

To assess the performance of local re-alignment with SRMA on a real-world dataset, a previously published whole-genome human cancer cell line (U87MG) was used (SRA009912.1) [13]. This dataset was aligned with BFAST (Blat-like Fast Accurate Search Tool) [7], which reported the original color sequence and color qualities accompanying each alignment. This allows local re-alignment to be performed in color space by adapting the existing two-base encoding algorithm to work on the variant graph structure [12,21]. The aligned sequences were then used for variant-calling with SAMtools [20], which also reported the zygosity of each call.

**Figure 2:**
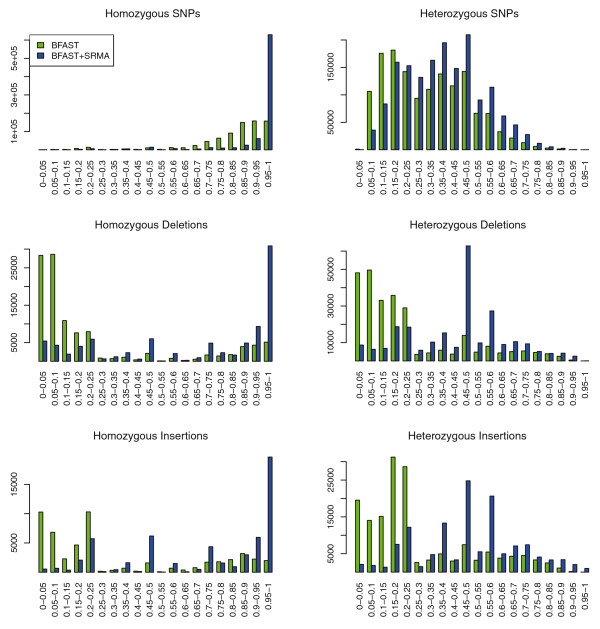
**Allele frequency distribution with local re-alignment of U87MG**. SRMA was applied to the alignments produced with BFAST of a human cancer cell line (U87MG; SRA009912.1). Variants were called with SAMtools before and after application of SRMA (see Materials and methods). Homozygous and heterozygous calls were examined independently using zygosity calls produced by SAMtools. The observed non-reference allele frequency for SNPs, deletions, and insertions are plotted for homozygous (left panels) or heterozygous variants (right panels). Ideally, non-reference allele frequencies for homozygous and heterozygous variants approach 1.0 and 0.5, respectively. The absolute counts of observed variants are plotted (y-axis) against non-reference allele frequency ranges (x-axis).

In the case of SNPs called from color space (two-base encoded) data, the decoded reads can be improperly decoded such that SNP positions have a reference allele bias, which is reflected in the original alignments. Thus, in order to assess if SRMA is improving the overall fraction of reads appropriately aligned, we analyzed in aggregate all variant positions to determine if the ratio of reference/variants at heterozygous positions is shifted towards the expected 50%. With respect to heterozygous-called variants, a binomial distribution centered around 0.5 frequency based on sampling/coverage is expected. The observed variant allele frequency after SRMA is substantially shifted towards this expected distribution (Figure 2). Similarly, at homozygous positions, the non-reference allele is substantially closer to 100% across observed variant positions for SNPs, deletions, and insertions (Figure 2). For example, the median allele frequencies for heterozygous SNPs, deletions, and insertions before SRMA were 0.404, 0.038, and 0.038, respectively, and after SRMA were 0.434, 0.538, and 0.328, respectively. This demonstrates the ability of SRMA to improve variant calling, especially for indels.

**Figure 3:**
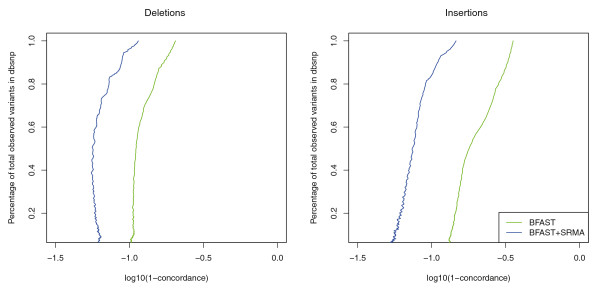
**dbSNP concordance before and after local re-alignment of U87MG**. SRMA was applied to the alignments produced with BFAST of a human cancer cell line (U87MG; SRA009912.1). Variants were called with SAMtools before and after application of SRMA (see Materials and methods). Deletions and insertions (indels) called within U87MG were compared with those indels reported in dbSNP (v129). An increasing minimum SNP quality filter was used to improve concordance (y-axis) while reducing the number of indels observed at dbSNP positions (x-axis). Using SRMA significantly reduced the discordance (one minus concordance) between observed indels at dbSNP positions.

To further examine the accuracy of the variant calls genome-wide, indels were compared to the known database of common variants found in dbSNP (dbSNP Build ID: 129) [22]. We sought to determine if the indel matches a previously observed indel in dbSNP, which is plotted as the discordance rate (one minus concordance; Figure 3). An indel was called concordant if the length of the called indel matched that of any indel in dbSNP within five bases. This 'wiggle' of five bases was used since the precise location of an indel relative to the reference is not always systematically and consistently described in dbSNP. SRMA improves the concordance between observed indels within the sequencing data and indels reported in dbSNP. The discordance rate of indels is inflated due to the lack of completeness within the variant databases, as well as artifacts introduced by tandem repeats, and artifacts related to the arbitrary position of indels relative to the reference in dbSNP. However, using similar metrics, SRMA measurably improves the concordance: greater than 99% of SNPs (data not shown) and greater than 90% of indels were concordant with dbSNP regardless of the stringency threshold applied.

**Figure 4:**
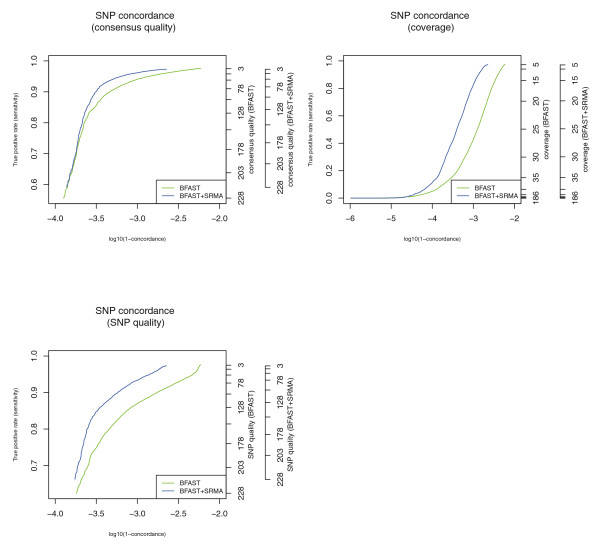
**SNP microarray concordance with known genotypes before and after local re-alignment of U87MG**. SRMA was applied to the alignments produced with BFAST of a human cancer cell line (U87MG; SRA009912.1). Heterozygous genotypes from an Illumina SNP microarray were compared with genotypes called from sequence data before and after application of SRMA (see Materials and methods). A minimum threshold on three different variant-calling metrics was applied, respectively, to improve the concordance (y-axis) while reducing the total number of SNP positions on the microarray that were called. Regardless of the metric, SRMA reduced the discordance (one minus concordance) of heterozygous SNPs reported by the SNP microarray and sequencing data.

To further assess the quality of SNP calls, heterozygous genotypes from an Illumina SNP microarray were compared with genotypes called from sequence data before and after application of SRMA to estimate SNP concordance. In Figure 4, the concordance between heterozygous calls and genotypes is reported after filtering positions using three metrics: consensus quality, base coverage, and SNP quality. A true positive occurred if a heterozygous SNP was called with the sequence data and genotyped as a heterozygote. A genotype was discordant if a heterozygous SNP was called with the sequence data but the genotype was called homozygous on the DNA microarray. For all metrics, local SRMA re-alignment reduces the discordance rate while preserving sensitivity. It is interesting to note that the discordance rate after SRMA approaches the assumed DNA microarray error rate, thus limiting further utility of this type of comparison.

**Figure 5:**
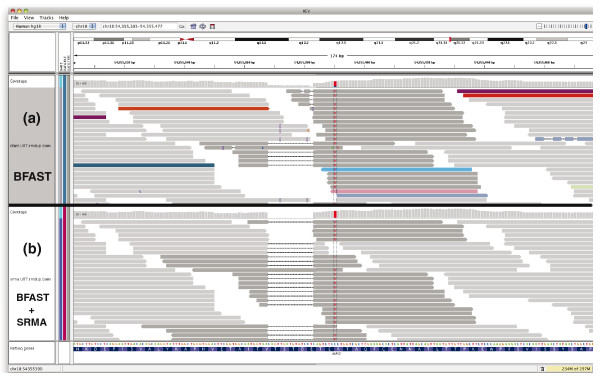
**A deletion and SNP in ALPK2 in U87MG**. SRMA was applied to the alignments produced with BFAST of a human cancer cell line (U87MG; SRA009912.1). **(a,b) **The resulting alignments from within the coding region of ALPK2 (chr18:54,355,303-54,355,477) are shown before applying SRMA (a) and after applying SRMA (b). In this haploid region, Sanger sequencing confirmed a 15-bp deletion and a C-to-T SNP eight bases downstream of the deletion. Panel (a) shows the difficulty of aligning sequence reads from a region with a large deletion and a SNP, as false variation is observed (SNPs and indels). Nevertheless, some reads in **(a) **(BFAST) do correctly observe the deletion and SNP, which are therefore included in the variant graph created by SRMA. After local re-alignment using SRMA **(b)**, the majority of the reads support the presence of the deletion and SNP, while false variation has been eliminated. The Integrated Genomics Viewer was used to view the alignments [30].

The variant calls of SRMA are improved genome-wide by SRMA, and several dramatic examples of sequence improvement can be demonstrated. For instance, a 15-bp deletion flanked by a nearby C-to-T SNP was observed in the coding sequence of ALPK2 in the original BFAST alignments of U87MG and was confirmed by Sanger sequencing. However, a large fraction of the original alignments did not contribute to the calling of this haploid event (Figure 5a), instead displaying spurious SNPs, deletions, and insertions. This nicely demonstrates the inherent difficulty of comparing a short read sequence to a reference sequence in the presence of variation and sequencing error, even though the short reads were all aligned to the correct location in the genome. After re-alignment with SRMA (Figure 5b), the majority of the reads support both the 15-bp deletion and SNP, while false variation has been virtually eliminated.

### Performance of local re-alignment

The running time and memory required by this re-alignment procedure is based on the number of start nodes as well as the complexity of the variant graph. More start nodes (larger *w*) will increase the number of paths examined. Furthermore, any variant within the graph will lead to a larger branching factor (nodes with multiple neighbors either upstream or downstream) and increase the number of paths examined. Highly polymorphic genomes will also increase the graph's complexity. The complexity of the graph is also influenced by the sequencing technology. For technologies that sequence DNA bases directly, sequencing errors that are indistinguishable from variants will thus be represented in the graph. The two-base encoded data produced by the ABI SOLiD system in practice tends to have fewer spurious variants. With such an encoding, it is more difficult to interpret sequencing error in the encoded color sequence in such a fashion as to produce base changes in the decoded base sequence. Nevertheless, without filtering using the *c *or *p *parameters, any observed base difference from an alignment will be included in the graph. Therefore, setting reasonable parameters for *c *and *p *beyond removing spurious variants is important to bound the number of search paths and make re-alignment computationally feasible. In practice, the settings used in our evaluations (*c *= 2 and *p *= 0.1) work well for human genome re-sequencing experiments

SRMA was run in a Map-Reduce framework using a cluster submission script (for Sun Grid Engine (SGE) or Portable Batch System (PBS) systems) provided with the SRMA distribution. The alignments to the reference genome were implicitly split into 1-Mb regions and processed in parallel on a large computer cluster; the re-alignments from each region were then merged in a hierarchical fashion. This allows for the utilization of multi-core computers, with one re-alignment per core, as well as parallelization across a computer cluster or a cloud. The average peak memory utilization per process was 876 Mb (on a single-core), with a maximum peak memory utilization of 1.25 GB. On average, each 1-Mb region required approximately 2.58 minutes to complete, requiring approximately 86.17 hours total running time for the whole U87MG genome. SRMA also supports re-alignment within user-specified regions for efficiency, so that only regions of interest need to be re-aligned. This is particularly useful for exome-sequencing or targeted re-sequencing data.

## Conclusions

Here we describe a novel local re-alignment algorithm, SRMA, which can significantly reduce the false positive variant detection rate with short-read next generation sequencing technology. While global sequence alignment examines each read independently, multiple reads aligned over a common position are highly correlated especially when a single diploid genome is being sequenced. SRMA uses these correlated alignments to build a limited graph structure that represents these alignments and their differences in compact form such that the alternative allele is more readily observed. The original reads are then re-aligned within a local coordinate window to improve the resulting alignments relative to the target genome rather than a reference genome.

Simulations of whole genome human re-sequencing data from both ABI SOLiD and Illumina sequencing technology were used to assess SRMA under simplified conditions in which the variant positions and alleles are known. SRMA was able to improve the ultimate variant calling using a variety of measures on the simulated data from two different popular aligners, BWA and BFAST. These aligners were selected based on their sensitivity to insertions and deletions since a property of SRMA is that it produces a better consensus around indel positions. The initial alignments from BFAST allow local SRMA re-alignment using the original color sequence and qualities to be assessed as BFAST retains this color space information. This further reduces the bias towards calling the reference allele at SNP positions in ABI SOLiD data, and reduces the false discovery rate of new variants. Thus, local re-alignment is a powerful approach to improving genomic sequencing with next generation sequencing technologies.

We note as well that while clearly demonstrating improvements in human genomic sequencing, more substantial improvements in variant discovery would be expected when a more distantly related genome is used as the reference. Currently, SRMA does not support enumerating over insertions or deletions caused by homopolymer errors that can be found in 454 data and other flow-based technologies. Nevertheless, similar to utilizing the original color sequence for ABI SOLiD data, the original flow-space data from 454 data could be used during re-alignment and represents future work. Incorporating known variants, for example from dbSNP, into the variant graph as a prior also represents future work. SRMA is publicly available under the GPL license at [23].

## Materials and methods

### Overview of SRMA

This method relies on short-read alignment algorithms to first align each read to a reference sequence [7-12]. After all reads are aligned, they are passed to SRMA for re-alignment. SRMA first builds a variant graph from these initial alignments. Once the variant graph is built, all reads are re-aligned to the variant graph. If the new alignment compared to the original is found, it is reported and annotated as being re-aligned by SRMA, otherwise the original alignment is reported. A novel aspect of this method is the process of building the variant graph iteratively for each genomic region, while reporting new alignments for each read initially aligned within that region. While *de novo *assembly (or re-assembly) algorithms report novel sequences without comparing the reads to a reference sequence, this method provides new improved alignments relative to a reference sequence improving downstream consensus calling. Iterative application of SRMA is possible, whereby further rounds of building a variant graph and read re-alignment are performed, but is not examined here.

### Creating a variant graph from existing alignments

Here we seek to use individual sequence reads to create a series of possible variant options that include the true variants present within the target genome being sequenced. Ultimately, the goal is to distinguish between true variants and sequencing errors genome-wide. Since, in the interest of novel mutation discovery, we must allow for all possible base positions being variant, as well as for an exponentially larger number of possible indels, we opt for an approach that creates a variant graph that includes all aligned reads at a given position in the genome prior to performing re-alignment. This graph is a compact mathematical representation of the initially determined alignments. Each alignment is represented as a path through the graph, although not every path through the graph corresponds to an actual alignment.

The variant graph is composed of nodes. Each node represents a DNA base at a specific position relative to the forward strand of the reference genome. Two nodes share an undirected edge if they are adjacent read bases in an existing alignment. For example, the variant graph of the reference sequence that is aligned perfectly to itself consists of one node per reference base, with edges connecting nodes that represent adjacent bases in the reference. In this case, the variant graph has one path. To properly order the nodes in the graph relative to the reference, each node is also assigned a position and an offset. The offset is non-zero only if the node represents an insertion relative to the reference. Insertions relative to the reference are given the reference position of the next non-inserted base with higher physical position on the forward strand, and with its offset set as the number of bases from the beginning of the insertion. Insertions at the same reference position can be combined by merging the paths that represent their longest common prefix and longest common suffix, respectively. A single nucleotide substitution would be annotated to have the same position as its relative reference base. In summary, nodes are described as three distinct types: reference, substitution, and insertion. A node's position, base, type, and offset are unique among all nodes in the graph and define a canonical ordering over all nodes in the graph.

**Figure 6:**
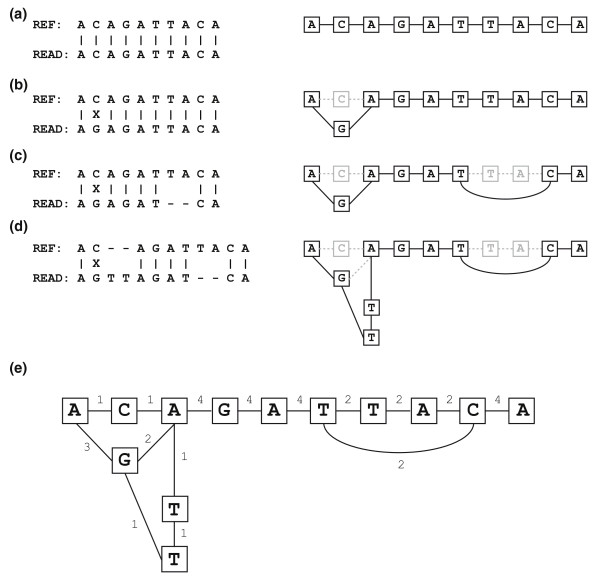
**The creation of a variant graph**. Four alignments (left) are successively used to create a variant graph (right). **(a) **An alignment of a read that matches the reference. The associated variant graph consists of nodes that represent each base of the read. **(b) **An alignment of a read with a base difference at the second position. The base difference adds a new node that is connected to the existing first and third node. **(c) **An alignment of a read that has a base difference and a deletion relative to the reference. A new edge connecting the sixth and ninth nodes is added to the graph. **(d) **An alignment of a read that has a base difference, a deletion, and an insertion relative to the reference. Two new nodes are added creating a path from the previously existing SNP at the second position to the reference base at the second position. **(e) **The resulting variant graph with each edge labeled with the number of alignment paths containing this edge.

Initially, the graph is empty. Bases that match the reference and variants are incorporated into the graph by adding new nodes and edges. Substitutions and insertions are represented as additional nodes in the graph. Deletions, on the other hand, are added as edges that connect nodes that have a positional difference greater than one. An example of creating a variant graph from four alignments is shown in Figure 6. The variant graph also stores the number of alignments that pass through each node and edge, corresponding to the coverage. This is useful for eliminating unlikely paths when performing re-alignment and will be discussed later.

### Alignment to a variant graph

Once the variant graph is constructed from all aligned reads, local re-alignment of the reads proceeds through a series of weighted steps to optimize the final alignments. The variant graph is not modified after re-alignment begins. A dynamic programming procedure is used to compare a read to the variant graph in a similar manner to the Smith-Waterman algorithm [24-27]. Each path through the graph represents a potential (new) alignment. All paths that begin within *w *base positions from the start of the existing alignment are considered as start nodes for a new alignment. A node in the graph is visited at most *w *times per re-alignment, even though every path reachable from a starting node is examined. Note that the direction of the paths through the graph match the direction implied by the strand of the original alignment. Therefore, the graph is a directed acyclic graph (DAG) during each local re-alignment, with a partial ordering imposed on the nodes as was explained earlier (position, base, type, and offset). All valid paths from the starting nodes can be efficiently examined using a breadth-first traversal using a heap data structure.

The heap stores nodes sorted by their partial order, the current path length, and the current alignment score, in that order; the path length and alignment score are also stored in the heap. Initially, the start nodes are added to the heap with a path length of one and an alignment score based on comparing the read's first base to the base represented by the start node. If the read base matches the start node base, then no penalty is added to the previous re-alignment score. Otherwise, a negative score based on the original base quality of the read is added to the previous re-alignment score to return the current re-alignment score. Other alignment scoring schemes are possible, but mismatched bases are scored using base quality since it has been shown to improve alignment quality [28].

The heap is polled while it is non-empty. Paths to the given node that have the same path length and a smaller alignment score can be removed (from the top of the heap) to remove suboptimal alignment paths. Paths to the same node but with different lengths result from differing start nodes, deletions, and insertions. This pruning step uses a dynamic programming procedure, where the best paths to and from the current node are assumed to be conditionally independent given their respective path lengths (number of read bases examined). Next, if the path length equals the length of the read, all of the bases in the read have been examined. The best (highest alignment score) complete path, if any, is compared to the current path and updated accordingly. Otherwise, the path is extended to each child (successor) of the given node. For each child node, the child node's base is compared to the corresponding base in the read (determined by the path length), with the alignment score modified as above. The child node, incremented path length, and updated alignment score are added to the heap. Once the heap is empty, the path with the best score is returned to give a new alignment. This new alignment may match or differ from the original alignment depending on the graph structure.

As observed during graph creation, the original alignment is represented as a path through the graph, and therefore will be reconsidered during re-alignment. In fact, the original alignment can be used to set a bound on the minimum re-alignment score. Since the alignment score implemented above decreases monotonically, any path with lower alignment score than the original alignment can be removed from the heap. If the original alignment is likely to be the best alignment after re-alignment, then this bound significantly reduces the practical running time of local re-alignment.

The entire variant graph does not need to be constructed before beginning re-alignment, but rather only nodes in the graph that are reachable from the starting nodes need be considered. Therefore, only original alignments that pass through any of these reachable nodes need to be included when creating the variant graph for a specific alignment. Thus, the variant graph can be dynamically built from previous read alignments, with nodes removed from the graph when no longer reachable from the next read re-alignment. This allows only a small local window of the variant graph to be explicitly built and kept in memory, significantly reducing memory requirements.

### Accounting for sampling and coverage

Two input parameters prune potential alignment paths through the graph: minimum node/edge coverage, and minimum edge probability. Given a minimum node/edge coverage *c*, only nodes observed in least *c *original alignments are considered. The minimum edge probability *p *considers the all edges through non-insertion nodes (that is, zero offset) at a given genomic position. The total number of observations *N *across all nodes with the same position (and zero offset) along with the minimum edge probability *p *is used to bound paths through edges incoming to nodes at that position. Suppose an incoming edge to a node is observed *n *times, then the edge is pruned if Pr(*x *≤ *n *| *N*) <*p*. This probability is modeled using the binomial cumulative distribution function under the assumption that two possible alleles (nodes) are possible at a given position:

$$\mathrm{Pr}\left(x\leq n|N\right)=\sum_{i=0}^{x}\left(\begin{array}{c} N\\ x \end{array}\right){\left(0.5\right)}^{x}{\left(0.5\right)}^{N-x}=\sum_{i=0}^{x}\left(\begin{array}{c} N\\ x \end{array}\right){\left(0.5\right)}^{N}$$

While this is a valid assumption if the genome has two copies of each chromosome (diploid), deviations from this do not greatly change the pruning strategy as both input parameters are used in conjunction with an *OR *logical relationship: a path through a node/edge is included if it passes one or both of the filters. Within high coverage locations, the former filter removes variants that occur by random chance due to sequencing error but is intended to remain sensitive to the detection of alleles that can be obscured by alignment ambiguities. In contrast, within low coverage regions, the former filter will overly penalize variants that were not observed due to insufficient sampling. Thus, the latter filter is designed to include variants in low coverage regions while strictly penalizing variants that do not occur frequently in high coverage regions. These parameters are important for both removing spurious variants and inclusively including potentially real variants in low coverage regions.

### Leveraging ABI SOLiD two-base encoded data

Special considerations need to be made to incorporate sequencing reads produced by the ABI SOLiD platform, which are generated in a two-base encoded form. When re-aligning such data to the variant graph, a modified version of the two-base encoded dynamic programming algorithm is used [12,21]. In this case, the decoded DNA sequence must exactly match the bases represented by a valid path in the variant graph. The re-alignment score is produced by using the original color sequence and quality scores and is calculated by comparing the original color in the color sequence with the expected color. The expected color is determined by encoding the two bases connected by the previously examined edge in the path, or encoding the known start adapter and first base in the path (for starting nodes). By constraining the decoded bases to match bases represented by nodes in the graph, the computational complexity of the original dynamic programming procedure is reduced to be equivalent to that of the base or nucleotide space sequence comparison.

### Simulated and empirical data

Two simulated datasets were created to evaluate SRMA, one simulating data from an Illumina sequencer, and one simulating data from an ABI SOLiD sequencer. A uniform 1% per-base error rate was used for the Illumina dataset, while a uniform 5% per-color read error rate was used for the ABI SOLiD dataset. In practice, sequencing error per read is not uniform, tending to be low at the 5' end (the beginning) of the sequence read and higher towards the end of each read, but that is not modeled here. The distance between the two ends of each paired end read was randomly drawn from a normal distribution of mean 500 bases and standard deviation of 50 bases. Polymorphisms were added to the simulated genome at a rate of 1/1000 with a 1/3 probability of being a homozygous variation. Insertions and deletions each accounted for 5% of all polymorphisms. The probability of an insertion or deletion extending beyond one base was 0.3 per extended base to simulate observed in/del distributions in the human genome. The whole-genome simulation program and subsequent accuracy evaluation can be found in the DNA Analysis (DNAA) package [29].

To empirically test the feasibility and utility of SRMA in a whole genome context, a previously published human whole-genome brain cancer dataset was obtained from the Sequence Read Archive (SRA009912.1) [13]. The original alignments were obtained, which were performed by BFAST [7], and retained the original color sequence and qualities to allow for color space local re-alignment [12,21]. The alignments from BFAST and SRMA were variant-called using the SOAP consensus model implemented in SAMtools (v.0.1.17) using the default settings [10,20]. The subsequent alignments were locally re-aligned with SRMA with variant inclusive settings (*c *= 2 and *p *= 0.1).

SAMtools reports three metrics for each variant position: SNP quality, consensus quality, and base coverage. The SNP quality is the Phred-scaled probability that the consensus is identical to the reference, while the consensus quality is the Phred-scaled likelihood that the called genotype is wrong. Typically, a minimum SNP quality filter can be used to reduce false positives while somewhat reducing sensitivity.

## Abbreviations

ABI: Applied Biosystems Inc.; BFAST: Blat-like Fast Accurate Search Tool; bp: base pair; BWA: Burrows Wheeler Alignment tool; dbSNP: Single Nucleotide Polymorphism Database; SNP: single nucleotide polymorphism; SRA: Sequence Read Archive; SRMA: Short-Read Micro re-Aligner.

## Authors' contributions

NH designed and implemented the algorithm, and drafted the manuscript. SFN drafted the manuscript. All authors read and approved the final manuscript.
